# Lipid analysis of *Eimeria* sporozoites reveals exclusive phospholipids, a phylogenetic mosaic of endogenous synthesis, and a host-independent lifestyle

**DOI:** 10.1038/s41421-018-0023-4

**Published:** 2018-05-22

**Authors:** Pengfei Kong, Maik J. Lehmann, J. Bernd Helms, Jos F. Brouwers, Nishith Gupta

**Affiliations:** 1 Department of Molecular Parasitology, Faculty of Life Sciences, Humboldt University, Berlin, 10115 Germany; 2 Department of Biochemistry and Cell Biology, Institute of Biomembranes, Utrecht University, Utrecht, 3584CM The Netherlands; 3 Present Address: Department of Life Sciences and Engineering, University of Applied Sciences, Bingen, 55411 Germany

## Abstract

Successful inter-host transmission of most apicomplexan parasites requires the formation of infective sporozoites within the oocysts. Unlike all other infective stages that are strictly intracellular and depend on host resources, the sporozoite stage develops outside the host cells, but little is known about its self-governing metabolism. This study deployed *Eimeria falciformis*, a parasite infecting the mouse as its natural host, to investigate the process of phospholipid biogenesis in sporozoites. Lipidomic analyses demonstrated the occurrence of prototypical phospholipids along with abundant expression of at least two exclusive lipids, phosphatidylthreonine (PtdThr) and inositol phosphorylceramide with a phytosphingosine backbone, in sporozoites. To produce them de novo, the parasite harbors nearly the entire biogenesis network, which is an evolutionary mosaic of eukaryotic-type and prokaryotic-type enzymes. Notably, many have no phylogenetic counterpart or functional equivalent in the mammalian host. Using *Toxoplasma gondii* as a gene-tractable surrogate to examine *Eimeria* enzymes, we show a highly compartmentalized network of lipid synthesis spread primarily in the apicoplast, endoplasmic reticulum, mitochondrion, and Golgi complex. Likewise, trans-genera complementation of a *Toxoplasma* mutant with the PtdThr synthase from *Eimeria* reveals a convergent role of PtdThr in fostering the lytic cycle of coccidian parasites. Taken together, our work establishes a model of autonomous membrane biogenesis involving significant inter-organelle cooperation and lipid trafficking in sporozoites. Phylogenetic divergence of certain pathways offers attractive drug targets to block the sporulation and subsequent transmission. Not least, our results vindicate the possession of an entire de novo lipid synthesis network in a representative protist adapted to an obligate intracellular parasitic lifestyle.

## Introduction

The protozoan phylum Apicomplexa comprises >6000 extant species of obligate intracellular parasites, many of which infect a wide range of organisms including livestock and humans^1^. Some of the prevalent and representative apicomplexan parasites of the mammalian hosts include *Plasmodium*, *Toxoplasma*, *Eimeria* and *Cryptosporidium*. The genus *Eimeria* represents the largest clade in the phylum Apicomplexa; it consists of >1800 species, most of which infect a specific host^2^. These parasites together impose a significant healthcare burden and socioeconomic impact globally. Most apicomplexans have complex lifecycles occurring in one or more host organisms. The natural lifecycle comprises asexual and sexual reproduction gyrating often between the primary (sexual) and secondary (asexual) hosts. Infective stages of apicomplexans are termed zoites, which are formed either after sexual (sporozoite) or asexual (tachyzoite, merozoite etc.) reproduction. The lifecycle begins with the transmission of sporozoites developing within sporulated oocysts following the completion of sexual reproduction. A single oocyst yields 4 (*Cryptosporidium*), 8 (*Toxoplasma*, *Eimeria*) or numerous (*Plasmodium*) sporozoites after sporogony. Unlike other infectious stages that parasitize corresponding host cells to ensure survival and reproduction, sporozoite development occurs extracellularly and does not involve intimate interactions with host cells.

The formation of apicomplexan zoites obliges considerable lipid biogenesis when parasites replicate inside a parasitophorous vacuole (PV) enclosed within a host cell, or within oocysts. Previous studies on *Toxoplasma* tachyzoites and *Plasmodium* merozoites have demonstrated that these parasites not only deploy host-derived precursors to synthesize the required phospholipids^3–9^, but also are competent in salvaging selected lipids from the sheltering host cells^10–12^. It remains enigmatic how freely developing sporozoites satisfy their phospholipid demands being outside the host milieu. Moreover, from a conceptual viewpoint, the sporozoite stage imparts an excellent model to evaluate the “actual” metabolic potential of otherwise host-dependent organisms. The sporozoite metabolism has nonetheless been a black box from any typical apicomplexan parasite, principally because it is challenging to attain sufficient amounts of sporozoites for downstream biochemical analyses from non-model hosts.

De novo synthesis of the main phospholipids commences with the assembly of lysophosphatidic acid (Lyso-PtdOH) and phosphatidic acid (PtdOH) using glycerol 3-phosphate (Glycerol-3P) and fatty acids (Figure S1). In prokaryotes, PtdOH is converted into CDP-diacylglycerol (CDP-DAG) that serves as a substrate to synthesize all phospholipids^13^. In eukaryotes, PtdOH functions as a precursor for both CDP-DAG and diacylglycerol (DAG), which subsequently drive syntheses of distinct phospholipid classes^13^. CDP-DAG is utilized to make phosphatidylinositol (PtdIns) and phosphatidylglycerol (PtdGro), while DAG enables the synthesis of phosphatidylcholine (PtdCho) and phosphatidylethanolamine (PtdEtn) via the CDP-choline or CDP-ethanolamine pathways, respectively. PtdEtn can also be made by decarboxylation of phosphatidylserine (PtdSer), which itself is either derived by a base-exchange reaction from PtdEtn or PtdCho (mammals) or produced by fusion of CDP-DAG and serine (yeast)^14^. In some eukaryotic cells, such as yeast and mammalian hepatocytes, PtdEtn can also be methylated to yield PtdCho. In addition to the aforementioned archetypal lipid network, apicomplexan pathogens have evolved many novel and often physiologically essential pathways. Some of them have originated by secondary endosymbiosis of their common ancestor with a red alga^15–19^. Recently, we have identified a unique lipid, termed phosphatidylthreonine (PtdThr), in *Toxoplasma gondii*, which is crucial for calcium homeostasis governing the lytic cycle^7,20,21^. Such divergent or parasite-specific lipid synthesis pathways offer therapeutic targets to selectively inhibit the parasite reproduction.

This study employed *Eimeria falciformis*, a monoxenous (single-host) parasite infecting mouse, to discern the metabolic design of sporozoites^22–24^. We reveal a highly compartmentalized network of lipid synthesis, enabling sporozoites to produce generic as well as exclusive phospholipids in a self-sustained manner. The natural presence of atypical lipids together with autonomous biogenesis and evolutionary divergence of certain pathways offer a unique opportunity to prevent the process of sporulation and thereby inter-host transmission.

## Results

### The lipid profile of *Eimeria* sporozoites differs markedly from *Toxoplasma* tachyzoites

**Fig. 1:**
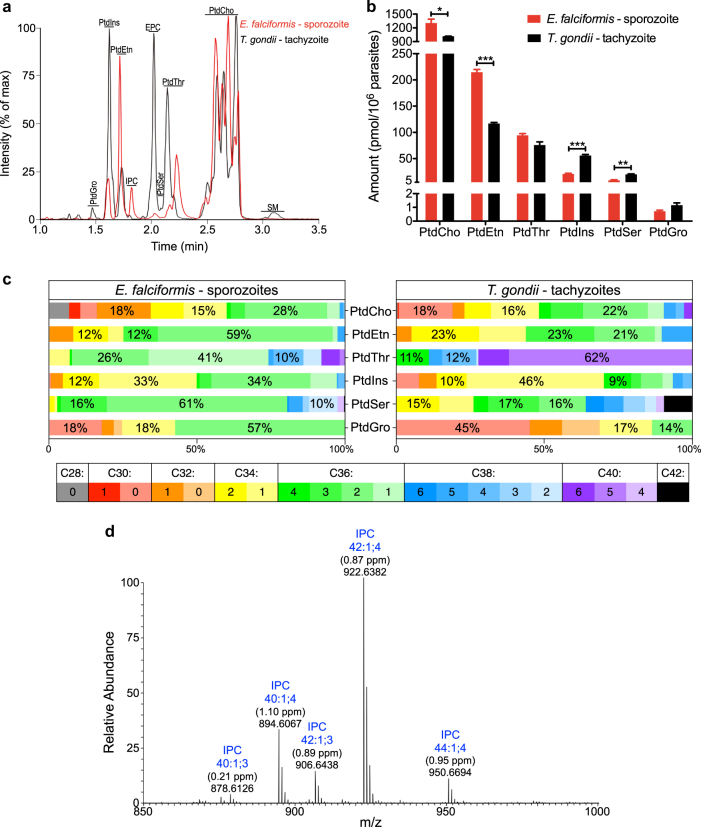
*E. falciformis* sporozoites and *T. gondii* tachyzoites share most phospholipid classes, but show a remarkably different composition of individual species. **a** Representative base peak chromatograms showing the retention times and relative intensities of lipid classes of *E. falciformis* sporozoites and *T. gondii* tachyzoites. **b** Amounts of major glycerophospholipids expressed in *E. falciformis* and *T. gondii*. The numerical values show the mean with SEM from 3 independent assays (*p < 0.05, **p < 0.01, ***p < 0.001). **c** Colored spectra comparing the composition of major glycerophospholipid species between the two parasitic stages. Color-coding shows the number of carbons and degree of saturation in acyl chains bound to indicated lipids, as confirmed by MS/MS. Percentages of the three most abundant species in each lipid class are shown on the corresponding bars. The data show the mean of 3 independent assays. Note that error bars are not depicted to avoid further complexity. **d** Average high-field orbitrap mass spectrum showing accurate masses during the elution of IPC, isolated from sporozoites of *Eimeria*. Peaks are annotated with the best database matches, together with the corresponding mass errors in parenthesis. In accordance with current nomenclature, the total number of carbon atoms (xx), the number of double bonds (yy), and the number of hydroxyl groups (zz) in the ceramide part of the molecule are depicted as “xx:yy;zz”. The corresponding structure of the most abundant species and its fragmentation in both positive and negative ion mode is given in Supplementary Figure S2

To examine the phospholipid composition of *E*. *falciformis* sporozoites, we isolated total lipids from purified parasites and performed high-performance liquid chromatography (HPLC). By this procedure, lipids were separated primarily based on their head-group (although some species resolution can be observed, Fig. 1a), thus eliminating any confusion between isobaric lipids from different classes. The total lipids extracted from the tachyzoites of *T*. *gondii*, which served as a comparative reference, were also analyzed alongside. The most abundant phospholipid detected in sporozoites was PtdCho (79.3%), followed by PtdEtn (13.1%), PtdThr (5.8%), PtdIns (1.3%), PtdSer (0.5%) and PtdGro (0.04%), whereas the dominant sphingolipid is inositol phosphorylceramide (IPC) (Fig. 1a). *T*. *gondii* tachyzoites shared similar phospholipid classes with *E*. *falciformis* sporozoites, but possessed ethanolamine phosphorylceramide (EPC) and sphingomyelin (SM) instead of IPC as the major sphingolipids, as also reported previously^7,25^ (Fig. 1a). The main PtdThr peak of *E*. *falciformis* appeared at a later retention time than the one of *T*. *gondii*, which indicated the presence of different species in these two parasites. Quantification of lipids based on calibration curves using standard lipids showed that *E*. *falciformis* sporozoites harbor more PtdCho and PtdEtn, but less PtdIns and PtdSer than *T*. *gondii* tachyzoites (Fig. 1b). Collectively, *Eimeria* contains significantly more phospholipids per cell than *T*. *gondii*, which is likely due to the bigger size of sporozoites than tachyzoites (10–12 vs. 7–8 μm)^24^.

Online tandem mass spectrometry (MS/MS) analysis of HPLC-eluted phospholipids revealed that the acyl chain compositions of different species were much more uniform in sporozoites compared to tachyzoites. The most abundant species in each phospholipid class of tachyzoites included PtdCho 36:2 (22%), PtdEtn 36:3 and 34:2 (both 23%), PtdThr 40:5 (62%), PtdIns 34:1 (46%), PtdSer 36:3 (17%), and PtdGro 30:0 (45%) (Fig. 1c). On the other hand, C36:2 (18:1/18:1) was the dominant species of most lipid classes in sporozoites, followed by shorter chains between C30 and C34 (Fig. 1c). PtdThr was the only exception with C36:1 (41%) and C36:2 (26%) as its first and second most abundant species (Fig. 1c). Moreover, when compared to other sporozoite phospholipids, PtdThr and PtdSer contained a higher proportion of species with long acyl chains C38–C42 (26% of PtdThr and 20% of PtdSer) and less of species with shorter acyl chains C28–C34 (7% of PtdThr and 3% of PtdSer). Collectively, these results show the presence of notably distinct lipid species in two related parasites, *E*. *falciformis* and *T*. *gondii*.

### *Eimeria* sporozoites express exclusive phytosphingosine species

The presence of IPC species in *Eimeria* sporozoites was not anticipated. Therefore, we investigated this lipid class further by high-field orbitrap MS to endorse its existence in *Eimeria* (Fig. 1d). A query for the accurate masses in the main lipid databases of Lipidbank (lipidbank.jp), LipidMaps (lipidmaps.org), and the Alex123 lipid calculator (https://www.ncbi.nlm.nih.gov/pubmed/24244551) found only IPC species as candidates with adequate mass accuracy (difference between theoretical and observed mass < 2 ppm). Furthermore, MS/MS analysis of the candidate IPC ions produced *m/z* 259.011 and *m/z* 241.022 as the primary fragment ions in the negative ionization mode, corresponding at the ppm-level to inositol phosphate after a loss of H_2_O (Figure S2A). Not least, the observed retention time of the lipid in question was 10–12 s longer than that of the matching glycerophospholipid class PtdIns (Fig. 1a). This is consistent with the observed retention time shifts for the sphingolipid classes found in *Toxoplasma* tachyzoites, where the sphingolipids EPC and SM elute 10–12 s after their related glycerophospholipid classes PtdEtn and PtdCho, respectively. Fragmentation of IPC species in positive mode revealed that IPC species contain four hydroxyl groups in the ceramide backbone (indicated by “4” suffix in the peak labeling of Fig. 1d). One of these hydroxyl groups is conjugated to the fatty acid moiety of the molecule as further fragmentation of the ceramide-like ion invariably lost one hydroxyl group together with the loss of the fatty acyl chain (Figure S2B). Likewise, IPC species containing only three hydroxyl groups (labeled “3” in Fig. 1d) in the backbone produced ion with *m/z* of 300.290. Hence, phytosphingosine was the only detected sphingoid base, and alpha-hydroxylation of the fatty acid was commonly observed in *Eimeria* sporozoites.

### *Eimeria* sporozoites encode an intricate network for de novo phospholipid biogenesis

Having identified major lipid classes expressed in sporozoites, we next performed a thorough bioinformatics search for the presence of corresponding enzymes in the *E*. *falciformis* genome (www.eupathdb.org)^23,26^. The query sequences included enzymes that have been characterized from various eukaryotic and prokaryotic organisms. We could identify a total of 21 enzymes potentially involved in phospholipid synthesis of *E*. *falciformis* (Table S1). Some enzymes, PtdGro phosphate phosphatase (PGPP), ethanolamine phosphotransferase (EPT) and PtdEtn methyltransferase (PEMT), could not be found in *E*. *falciformis*. We also compared the repertoire of lipid synthesis genes present in *E*. *falciformis* with other parasitic protists. Our results showed that three coccidian parasites *E*. *falciformis*, *T*. *gondii* and *Neospora caninum* shared similar inventory, which was bigger and more intricate than other apicomplexan (*Plasmodium* and *Cryptosporidium*) and kinetoplastid (*Trypanosoma* and *Leishmania*) parasites (Table S1). For example, *Eimeria*, *Toxoplasma* and *Neospora* all possess a PtdThr synthase (PTS), which is absent in other parasites. Likewise, many enzymes occur as two or even three distinct isoforms in coccidians, e.g., we detected three paralogs for DAG kinase (DGK) and choline/ethanolamine phosphotransferase (CEPT). In addition, we found 2 isoforms for PtdSer decarboxylase (PSD) and each of the enzymes synthesizing PtdOH (glycerol-3P acyltransferase or G3PAT; Lyso-PtdOH acyltransferase or LPAAT) and CDP-DAG (CDP-DAG synthase or CDS).

We were able to clone and experimentally annotate the full-length sequences of 19 enzymes using the RNA isolated from *Eimeria* sporozoites, which confirmed the transcription of nearly all lipid synthesis genes (Figure S3). The two open reading frames encoding for *Ef*DGK3 and *Ef*CEPT3 could not be amplified from the parasite RNA. Primary structures of the cloned enzymes revealed that all of them harbor intact catalytic domains with conserved residues required for substrate/cofactor binding, as exemplified for PtdSer synthase (PSS) and PTS (Figure S4). Most proteins except for LPAAT2, DGK1/2, PtdGro phosphate synthase (PGPS), cardiolipin synthase (CLS), and PSD1/2 harbor one or more defined transmembrane regions (Figure S3), signifying their membrane-binding nature. Notably, G3PAT1, DGK2, and PSD2 contain a predicted (secretory) signal peptide at their N termini. Equally, the N termini of PGPS, CLS and PSD1 comprise a mitochondrial targeting peptide. Together, the results suggest expression of an almost complete network for de novo synthesis of major phospholipids in *Eimeria* sporozoites.

### Lipid synthesis in *E. falciformis* is a phylogenetic mosaic of divergent pathways

**Fig. 2:**
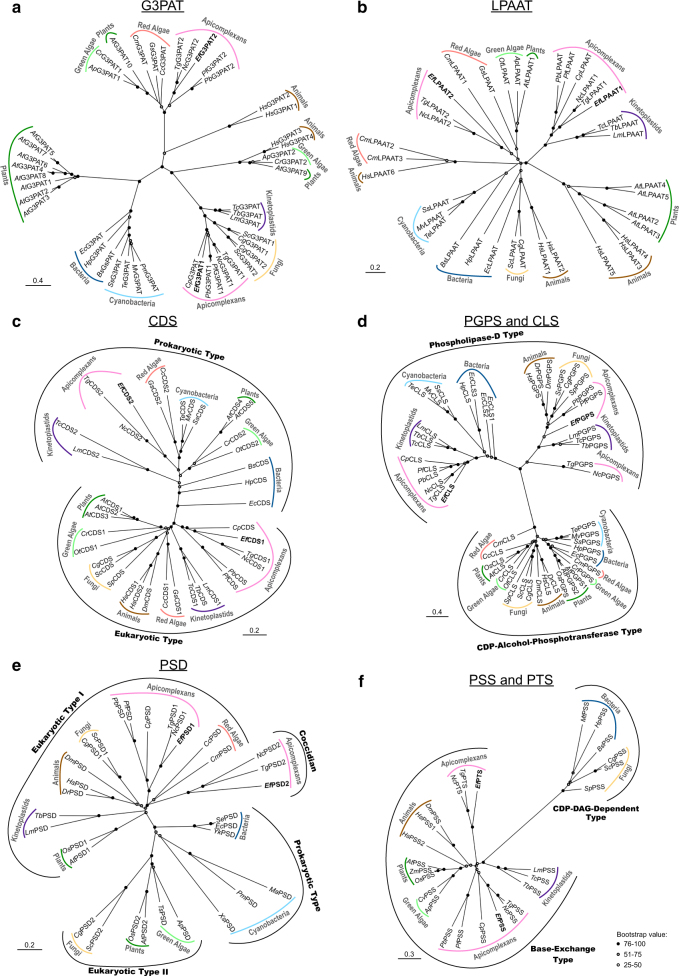
Enzymes of lipid synthesis in *E. falciformis* show diverse origins. Phylogenetic trees depicting the evolutionary relationships of G3PAT1/2 **a**, LPAAT1/2 **b**, CDS1/2 **c**, PGPS/CLS **d**, PSD1/2 **e**, and PSS/PTS **f** enzymes from *E. falciformis* with respective orthologs from varied organisms representing the major trees of life. Each tree represents the single most parsimonious tree constructed by Neighbor Joining methods. Branch support was estimated by 100 bootstrap replicates. Circles on the branches indicate the bootstrap values for parsimony. Scale bar represents the number of substitutions per amino-acid site. Sequence information including accession numbers and the organism names are described in Table S2

We performed phylogenetic clustering of selected enzymes of lipid biogenesis in *Eimeria* to determine their evolutionary origins. Specifically, G3PAT, LPAAT, CDS, PGPS/CLS, PSD, and PSS/PTS were subjected to cladogram analysis with respective orthologs from the major domains of the tree of life (Fig. 2). *Ef*G3PAT1 and *Ef*LPAAT1 segregated with corresponding homologs from other protozoan parasites, whereas *Ef*G3PAT2 and *Ef*LPAAT2 clustered with homologs from algae and plants (Fig. 2a, b). Similar phylogenetic patterns also applied to *Ef*CDS1 and *Ef*CDS2, of which the latter grouped with CDSs from not only algae and plants but also with orthologs from cyanobacteria (Fig. 2c). In the PtdGro and cardiolipin synthesis pathway, two enzymes (*Ef*PGPS and *Ef*CLS) were identified in *E*. *falciformis*, both encompassing the classic duplicated phospholipase D-like domains (Figure S3). *Ef*PGPS and *Ef*CLS clustered together with their protozoan homologs, as well as PGPSs from animals/fungi and prokaryotic CLSs, forming the phospholipase-D-type clade (Fig. 2d). The remaining PGPS and CLS sequences belong to the CDP-alcohol-phosphotransferase-type clade. The two PSDs from *E*. *falciformis* (*Ef*PSD1 and *Ef*PSD2) also grouped in different clades. *Ef*PSD1 orthologs are conserved across phyla forming a eukaryotic-type-I clade. However, *Ef*PSD2-type proteins could only be detected in closely related coccidian parasites (Fig. 2e). Phylogenetic analysis of *Ef*PSS and *Ef*PTS demonstrated them as being the base-exchange-type enzymes unlike the CDP-DAG-dependent counterparts of PSS present in bacteria and fungi. Again, *Ef*PSS orthologs are present across the domains of life, whereas *Ef*PTS-type proteins were found only in selected coccidian parasites (Fig. 2f). Collectively, these data reveal a surprising occurrence of fairly divergent enzymes in *Eimeria*, which have likely been repurposed to serve the parasitic lifecycle.

### Enzymes of *Eimeria* lipid synthesis show compartmentalized distribution

**Fig. 3:**
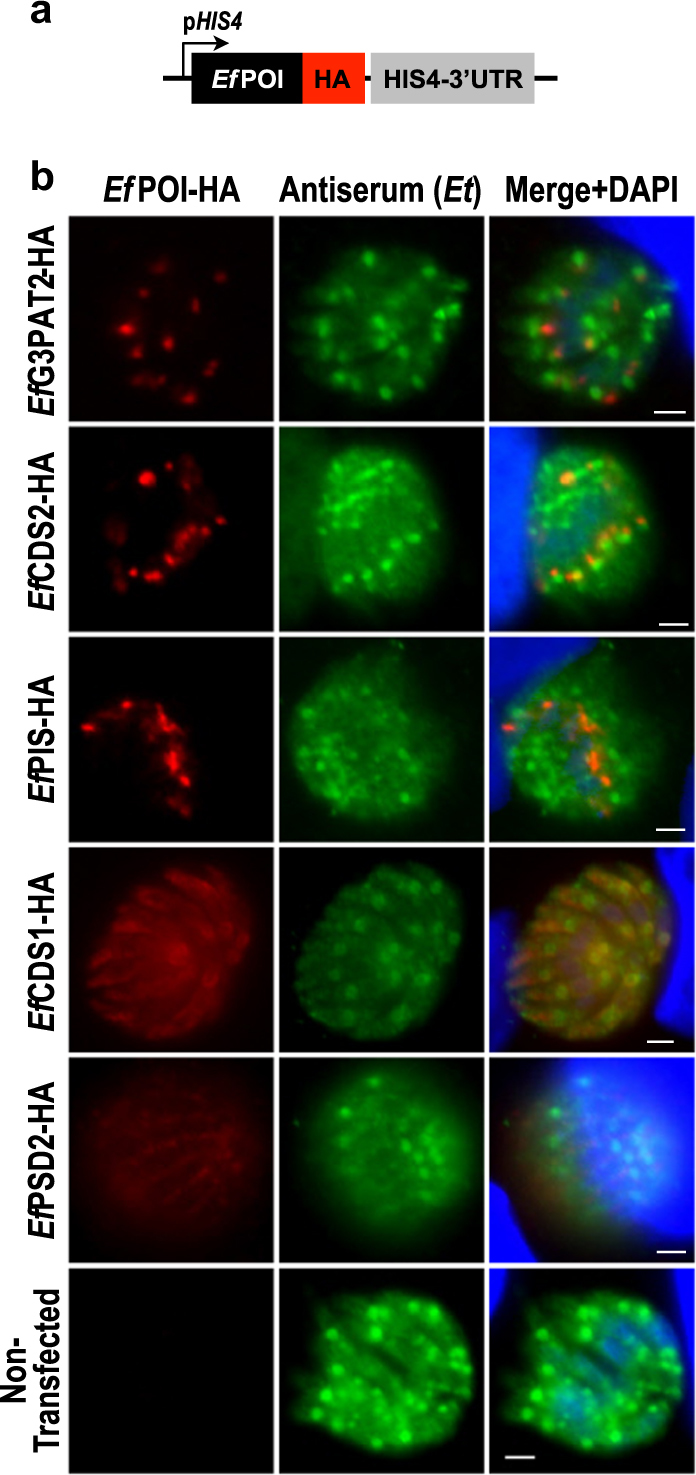
Localization of selected enzymes in *E. falciformis* merozoites developing within host cells. **a** Schematics of the expression cassette used to express a protein of interest in *E. falciformis* (*Ef*POI) under the control of *Ef*HIS4 promoter and 3’UTR. **b** Immunofluorescence images of transgenic merozoites expressing *Ef*G3PAT2-HA, *Ef*CDS2-HA, *Ef*PIS-HA, *Ef*CDS1-HA, and *Ef*PSD2-HA. Plasmid constructs harboring the indicated ORFs were transfected into sporozoites followed by infection of HFF cells, and immunostaining with anti-HA antibody and *E. tenella* antiserum cross-reactive to *E. falciformis* (40–42 h post infection). Non-transfected control parasites exhibited no apparent staining with anti-HA antibody. Scale bars: 2 μm

Next, we determined subcellular distribution of aforementioned proteins from *E*. *falciformis*. A parallel aim was also to establish an initial genetic manipulation system for *E*. *falciformis*. We first overexpressed selected enzymes tagged with a C-terminal HA epitope in *E*. *falciformis* under the control of Histone 4 (*Ef*HIS4) promoter and matching 3′UTR (Fig. 3a). Transgenic parasites along with non-transfected control sample were visualized by indirect immunofluorescence assays (Fig. 3b). We were able to localize several albeit not all proteins, which exhibited distinct subcellular distributions. Algal-type *Ef*G3PAT2, prokaryotic-type *Ef*CDS2, and *Ef*PIS showed specific punctate signals located within each merozoite enclosed in schizonts. Eukaryotic-type *Ef*CDS1 displayed faint staining inside merozoites, while coccidian-specific *Ef*PSD2 appeared to be outside merozoites but within schizonts.

k

**Fig. 4:**
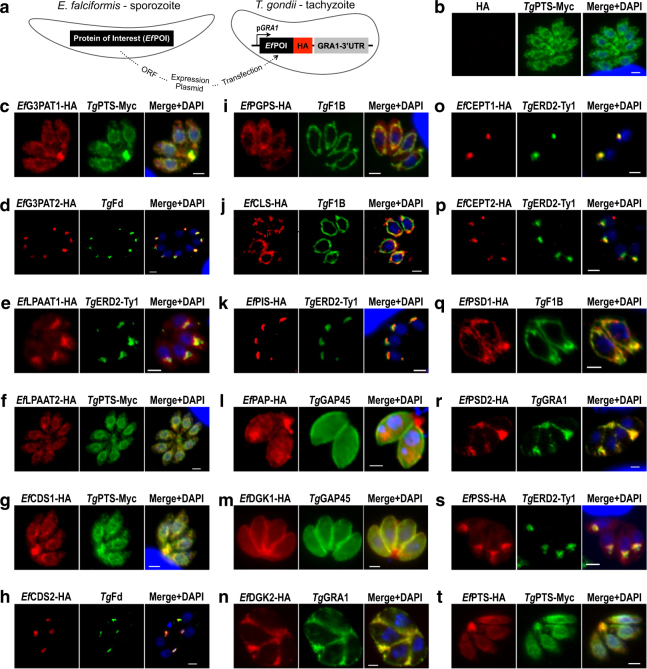
Ectopic expression of *Eimeria* enzymes in *Toxoplasma* tachyzoites reveals a compartmentalized distribution of lipid biogenesis. **a** Scheme showing expression of *E. falciformis* ORFs into tachyzoites of *T. gondii* under the control of *Tg*GRA1 promoter and 3’UTR. **b** Tachyzoites expressing *Tg*PTS-Myc only showing the absence of HA signal (a representative control). **c**–**t** Immunofluorescence images of the transgenic tachyzoites expressing *Ef*G3PAT1/2-HA, *Ef*LPAAT1/2-HA, *Ef*CDS1/2-HA, *Ef*PGPS-HA, *Ef*CLS-HA, *Ef*PIS-HA, *Ef*PAP-HA, *Ef*DGK1/2-HA, *Ef*CEPT1/2-HA, *Ef*PSD1/2-HA, *Ef*PSS-HA, and *Ef*PTS-HA. Constructs harboring indicated ORFs were transfected into tachyzoites followed by staining with anti-HA and Alexa594 antibodies (20–24 h post infection). Subcellular localization of each enzyme was confirmed by co-localization with respective organelle markers, including *Tg*PTS-Myc for ER, *Tg*Fd for apicoplast, *Tg*ERD2-Ty1 for Golgi, *Tg*F1B for mitochondrion, *Tg*GAP45 for the parasite periphery, and *Tg*GRA1 for the parasitophorous vacuole. Scale bars: 2 μm. Images shown are representative of at least three independent transfections. Note that the localization of certain enzymes might be subject to artifact due to ectopic overexpression in *T. gondii*. A schematized model showing subcellular distribution of all indicated enzymes can be seen in Fig. 7

The lack of appropriate organelle markers and sustained in vitro culture of *E*. *falciformis* prevented us from interpreting localization results. We therefore performed ectopic overexpression of selected enzymes in tachyzoites of *T*. *gondii*. Transgenic tachyzoites expressing *Eimeria* enzymes with a C-terminal HA tag were co-localized with different organelle markers (Fig. 4a). Tachyzoites expressing *Tg*PTS-Myc only were included as a control (Fig. 4b), which showed no background HA signal. Among transfected samples, *Ef*G3PAT1-HA and *Ef*CDS1-HA were targeted to the endoplasmic reticulum (ER; Fig. 4c, g), whereas *Ef*G3PAT2 and *Ef*CDS2 were found in the apicoplast (Fig. 4d, h), which corresponds with the phylogenetic origin of both isoforms and the evolutionary trace of this organelle. *Ef*LPAAT1-HA was expressed primarily in the Golgi complex with a faint staining in the ER (Fig. 4e). Surprisingly, *Ef*LPAAT2 was located mainly in the ER notwithstanding its algal origin (Fig. 4f), indicating an incomplete apicoplast pathway and potential transport of lyso-PtdOH and PtdOH between ER/Golgi and apicoplast. Enzymes of the PtdGro and cardiolipin pathway, *Ef*PGPS and *Ef*CLS, were expressed in the mitochondrion (Fig. 4i, j), whilst the PtdIns synthase (*Ef*PIS) was localized in the Golgi complex (Fig. 4k). The results suggest a transfer of CDP-DAG from the apicoplast/ER to the mitochondrion for the syntheses of PtdGro and cardiolipin, and to the Golgi network for the synthesis of PtdIns.

In eukaryotes, PtdOH is not only used for CDP-DAG production, but also for the synthesis of DAG, which is regulated by two reactions catalyzed by PtdOH phosphatase (PAP) and DGK (Figure S1). *Ef*PAP-HA displayed punctate intracellular distribution in cytomembranes (Fig. 4l), while *Ef*DGK1-HA localized in the parasite periphery (Fig. 4m) and *Ef*DGK2-HA was secreted into the PV (Fig. 4n). DAG serves as a co-substrate for CEPT to synthesize PtdCho and PtdEtn via the Kennedy pathways (Figure S1). Unexpectedly, *Ef*CEPT1 and *Ef*CEPT2 were expressed in the Golgi complex (Fig. 4o, p). PtdEtn can also be generated by decarboxylation of PtdSer (Figure S1). *Ef*PSD1 and *Ef*PSD2 were targeted to the mitochondrion and PV, respectively (Fig. 4q, r). Consistent with localization results, all isoforms residing in the mitochondrion (*Ef*PGPS, *Ef*CLS, and *Ef*PSD1) and PV (*Ef*DGK2 and *Ef*PSD2) harbored targeting motifs at the N termini (mitochondrial or secretory; Figure S3). PtdEtn and/or PtdCho are used to produce PtdSer and PtdThr via base-exchange reactions catalyzed by PSS and PTS, respectively, both of which were detected in the parasite ER with a strong signal of *Ef*PSS in the Golgi network (Fig. 4s, t). Taken together, our localization studies demonstrate an assorted subcellular expression of *Eimeria* enzymes in merozoites of *E*. *falciformis* as well as in tachyzoites of *T*. *gondii*.

### Trans-genera expression of *Ef*PTS rescues the lytic cycle of the Δ*tgpts* mutant

**Fig. 5:**
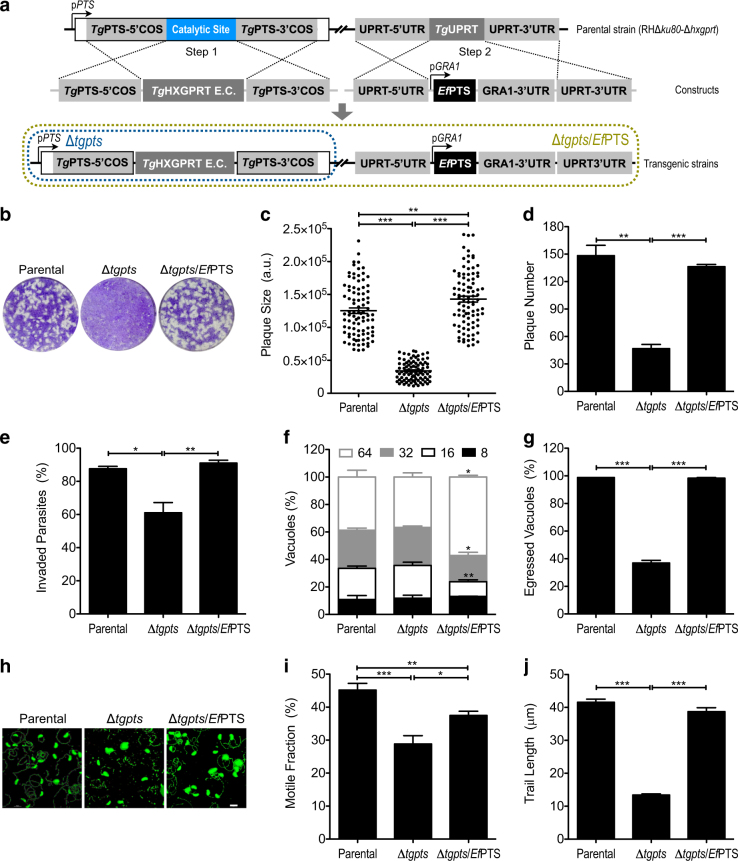
*Ef*PTS can complement all phenotypic defects in the PTS mutant of *T. gondii*. **a** Schematics illustrating a targeted disruption of the *Tg*PTS gene (Step 1), and subsequent insertion of *Ef*PTS expression cassette at the *TgUPRT* locus by negative selection (Step 2). The Δ*tgpts* mutant was created by Arroyo-Olarte RD et al., and found defective in invasion, egress and motility, but not in replication^7^. The complemented (Δ*tgpts*/*Ef*PTS) strain was made by expressing *Ef*PTS in the Δ*tgpts* mutant under the control of *Tg*GRA1 elements. **b**–**d** Plaque assays showing the phenotypic complementation of the Δ*tgpts* mutant by *Ef*PTS. The indicated strains were examined for their ability to recapitulate successive lytic cycles in HFF cells by plaque sizes (**c**) and numbers (**d**). 90 plaques of each strain were measured using the ImageJ suite. **e**–**g** Invasion, replication, and egress assays using the parental, Δ*tgpts* and Δ*tgpts*/*Ef*PTS strains. Invasion rates of 1500–2500 parasites of each strain are presented. A total of 200–350 vacuoles were numerated for the number of replicating parasites (**f**) or for natural egress (**g**). **h** Images demonstrating the gliding motility trails of specified strains, as visualized by immunostaining of *Tg*SAG1 protein. Scale bar: 10 μm. 100–120 parasites of each strain were scored for the motile fraction (**i**) and trail length (**j**). Numerical values in all graphs show the means with SEM from three independent assays (*p < 0.05, **p < 0.01, ***p < 0.001). COS, crossover sequence; E.C., expression cassette; a.u., arbitrary unit

Next, we tested the functionality of *Ef*PTS, a novel coccidian-specific enzyme, in a mutant of *T*. *gondii* lacking PTS expression. Our recent work has shown that the disruption of the PTS catalytic residues in tachyzoites (Fig. 5a—Step 1 and Figure S4) ablates the synthesis of PtdThr, which in turn compromises the calcium-dependent gliding motility, invasion and egress, leading to impairment of the lytic cycle^7,20,21^. The Δ*tgpts* mutant therefore not only offered a tool to test the catalytic function of *Ef*PTS, but also allowed us to assess the physiological significance of specific PtdThr species in tachyzoites (see below). The *Ef*PTS protein governed by p*GRA1* elements was expressed at the uracil phosphoribosyltransferase (*UPRT*) locus in the Δ*tgpts* mutant (Fig. 5a—Step 2). The eventual complemented strain (Δ*tgpts*/*Ef*PTS) was subjected to phenotypic assays to elucidate the biological impact of *Ef*PTS expression. The Δ*tgpts* mutant formed significantly smaller (−73%) and fewer (−69%) plaques compared to the parental strain in plaque assay, confirming the earlier work^7^. Quite notably, *Ef*PTS completely restored the lytic cycle of the PTS mutant (Fig. 5b-d). In-depth phenotyping of the Δ*tgpts* strain revealed an evident impairment in its invasion, egress and motility, but not in replication (Fig. 5e-j). The parasite invasion, egress and gliding motility were reinstated in the complemented strain. Remarkably, ectopic expression of *Ef*PTS also promoted intracellular proliferation of the mutant (Fig. 5f). These assays confirm that *Ef*PTS can compensate for the loss of its counterpart in tachyzoites.

### *Ef*PTS can restore the loss of tachyzoite-specific PtdThr species in the Δ*tgpts* mutant

**Fig. 6:**
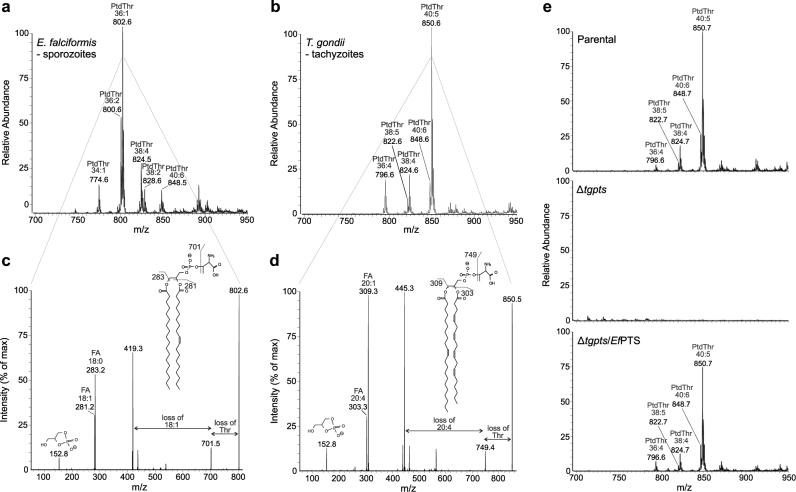
*Ef*PTS can produce tachyzoite-specific PtdThr species in the PTS knockout strain of *T. gondii*. **a**, **b** MS analysis of PtdThr isolated from the sporozoites of *E. falciformis* or from the tachyzoites of *T. gondii*. Lipid species were identified by their product spectra in the negative ionization mode. Note that the dominant PtdThr species in *Eimeria* harbor C36:1/C36:2 acyl chains in contrast to C40:5 in *Toxoplasma*. **c**, **d** MS/MS spectra of the major PtdThr peaks (*m/z* 802.6 for *E. falciformis* and *m/z* 850.6 for *T. gondii*). Acyl chains were identified as 18:0/18:1 (C36:1) and 20:1/20:4 (C40:5). **e** MS analysis of lipids isolated from the parental, Δ*tgpts* and Δ*tgpts*/*Ef*PTS strains of *T. gondii*. The data show a major (40:5) and several minor (40:6, 38:4, 38:5, and 36:4) PtdThr species in the parental strain, which are all absent in the Δ*tgpts* mutant. The loss of all indicated lipid species is restored by expression of *Ef*PTS in the Δ*tgpts*/*Ef*PTS strain

As mentioned above, the composition of PtdThr species were notably different between *E*. *falciformis* sporozoites and *T*. *gondii* tachyzoites (Fig. 1c). The most common PtdThr species in sporozoites was C36:1 (41%), followed by C36:2 (26%), C38:4 (10%), C34:1 (7%), C40:6 (6%), and C38:2 (6%) (Fig. 1c, 6a). In tachyzoites, however, all detected PtdThr species contained polyunsaturated fatty acid chains, including C40:5 (62%), 38:4 (12%), 36:4 (11%), 40:6 (10%), and 38:5 (4%) (Fig. 1c, 6b). The MS/MS analysis identified 18:0/18:1 and 20:1/20:4 as the most prevalent PtdThr species in *Eimeria* sporozoites and in the parental tachyzoites of *T*. *gondii*, respectively (Fig. 6c, d). As expected, lipidomic analysis of the Δ*tgpts* strain confirmed the lack of all PtdThr species (Fig. 6e). Surprisingly, complementation of the PTS mutant with *Ef*PTS fully restored the PtdThr content with the same species as present in the parental tachyzoites, but not with those expressed in *Eimeria* sporozoites. Interestingly, *Ef*PTS does not produce detectable amounts of 18:0/18:1 PtdThr in tachyzoites even though these fatty acids are readily available (Fig. 1c), suggesting a specific need of PtdThr containing polyunsaturated acyl chains (mainly 20:1/20:4) for the lytic cycle of *T*. *gondii*. Besides, because the potential donor species of PtdCho and PtdEtn with 20:1/20:4 are not detectable in *T*. *gondii*, synthesis of such PtdThr species is likely to occur predominantly by acyl chain remodeling of lipids.

### Sphingolipid synthase from *E. falciformis* is a plant-like protein located in the Golgi/ER network

Finally, we focused on characterizing yet-another enzyme underlying the synthesis of an exclusive lipid in *E*. *falciformis*, namely plant-like sphingolipids. Phylogenetic analysis revealed a distinct clade of sphingolipid synthase (SLS) from *E falciformis* along with homologs from arabidopsis and apicomplexan parasites (Figure S5A). SLS from kinetoplastid parasites, SM \synthase (SMS) from humans/animals, as well as IPC synthase (IPCS) from red algae and fungi formed their own individual clusters. Immunolocalization of C-terminally HA-tagged *Ef*SLS in tachyzoites of *T*. *gondii* exhibited a weak perinuclear staining (apparently in the ER) along with a notably strong signal at the anterior end of nucleus corresponding to the Golgi network (Figure S5B). Indeed, *Tg*ERD2, a known marker of the Golgi complex, co-localized with *Ef*SLS. We next performed functional complementation of a previously reported conditional yeast mutant, in which the promoter of endogenous IPC synthase has been replaced by a galactose-inducible element^27^. Consequently, the strain grows only in the presence of galactose but not glucose. As expected, the growth of the mutant under non-permissive condition could be restored by the *Sc*IPCS (aka *Sc*AUR1), but not by the empty vector (Figure S5C). *Tg*SLS also rescued the growth of the strain in glucose medium, confirming the expression of a catalytically competent enzyme. *Ef*SLS, however, failed to amend the growth defect under non-permissive condition, which may be due to functional divergence of lipid classes made by *Ef*SLS when compared to *Sc*IPCS and *Tg*SLS.

## Discussion

**Fig. 7:**
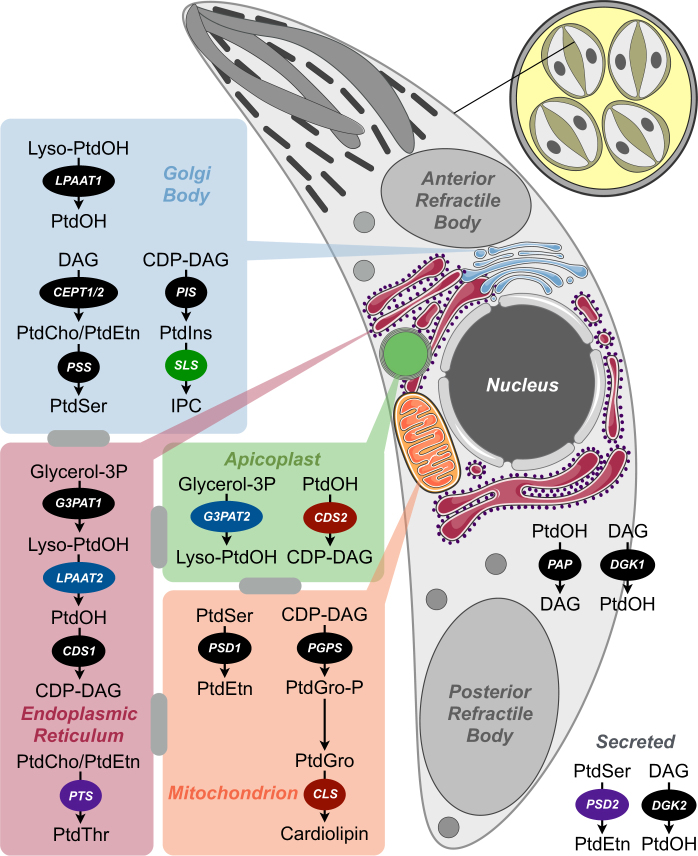
Compartmentalized network of phospholipid synthesis and underlying inter-organelle trafficking in *E. falciformis* sporozoites. The illustration reveals the pathways that have been identified based on expression and localizations of the enzymes in this study. Shown on the upper right corner is a sporulated oocyst enclosing four sporocysts, each with two sporozoites. Pathways located in individual organelles are zoomed in. The gray bars between indicated organelles show possible inter-organelle trafficking of phospholipids and precursors. The prokaryotic-type (*Ef*CDS2 and *Ef*CLS), algal-type (*Ef*G3PAT2 and *Ef*LPAAT2), plant-type (*Ef*SLS), and coccidian-specific (*Ef*PTS and *Ef*PSD2) enzymes are in red, blue, green, and purple backgrounds, respectively. Note that *Ef*LPAAT1 and *Ef*PSS also reveal a weak ER signal in addition to the dominant Golgi localization, as shown in Fig. 4

This study examined the phospholipid composition and the enzymatic network of lipid biogenesis in sporozoites of *E*. *falciformis*, which express all common eukaryotic glycerophospholipids, such as PtdCho, PtdEtn, PtdIns, PtdSer, and PtdGro (Fig. 7). The relative proportion of these lipids is similar to what has been described in various lifecycle stages of other protozoan parasites, including *T*. *gondii*^25,28^, *Plasmodium falciparum*^8,29–31^, *Trypanosoma brucei*^32,33^, *Trypanosoma cruzi*^34,35^, and *Leishmania donovani*^36,37^. Lipid species profiles in *Eimeria* sporozoites are quite noteworthy however; for instance, glycerophospholipids are composed of primarily saturated and monounsaturated fatty acid chains that are in most species not longer than C18. Among them, the most abundant acyl chains are C18:0 and C18:1, which form C36:1 (18:0/18:1) and C36:2 (18:1/18:1) as the dominant species for most glycerophospholipids. In addition, we show the natural occurrence of PtdThr in *Eimeria* sporozoites. PTS could only be found in the coccidian parasites (*E*. *falciformis*, *T*. *gondii*, *N caninum*), suggesting the evolution of PtdThr to enable a specialized function that could not be satisfied by generic phospholipids. Accordingly, expression of *Ef*PTS in the PTS mutant of *T gondii* produces PtdThr species with tachyzoite-specific fatty acids (mainly 20:1/20:4). The occurrence of distinct lipid species therefore appears to be critical for the development of individual stages.

Sphingophospholipids are another major class of lipids in eukaryotic cells. The main sphingophospholipid in fungi and plants is IPC^38–40^, while SM and EPC are present in mammals^41^. *T. gondii* tachyzoites also possess EPC and SM, confirming an earlier study^25^. Previous work in *T*. *brucei* have revealed the presence of IPC and SM in the procyclic stage of the parasite, as well as SM and EPC in the bloodstream stage^42^, indicating a highly stage-specific biogenesis of sphingophospholipids in this parasite. Here, we identified IPC as the prime sphingophospholipid in *E*. *falciformis* sporozoites, whose sphingoid backbone consisted exclusively of phytosphingosine (t18:0). In contrast, mammalian cells contain sphingosine (d18:1)-based backbones. This suggests that *Eimeria* is indeed capable of synthesizing the complete IPC lipid class de novo. Since IPC synthesis has no equivalent in mammalian cells, SLS enzymes with IPC synthase activity are considered as potential drug targets to treat protozoan infection^27,43,44^.

In *Toxoplasma* and *Plasmodium*, short saturated acyl chains (C14:0, C16:0) are produced through the prokaryotic-type (type II) fatty acid synthase pathway in the apicoplast^45,46^. These acyl chains are then exported to the ER, where they are modified by fatty acid elongase pathway to generate longer saturated and unsaturated fatty acids^45^. They are coutilized with Glycerol-3P to produce Lyso-PtdOH and PtdOH by sequential catalytic actions of G3PAT and LPAAT. PtdOH is then utilized by CDS and PAP to synthesize CDP-DAG and DAG, respectively. We found a fungal-type *Ef*G3PAT1 in the ER and an algal-type *Ef*G3PAT2 in the apicoplast (Fig. 7), which is consistent with recent reports on localizations of their homologs in *Toxoplasma* and *Plasmodium*^16,18,19,47^. Besides, *E*. *falciformis* expresses two paralogs of LPAAT and CDS. The eukaryotic-type LPAAT and CDS including *Ef*LPAAT1 and *Ef*CDS1, exist in the ER or ER/Golgi complex in all examined parasitic protists^16,48–51^. Homologs of algal-type *Ef*LPAAT2 and prokaryotic-type *Ef*CDS2 occur only in coccidians, which implies a loss of these two enzymes during the evolution of other parasites (Table S1). *Ef*CDS2 is likely localized in the apicoplast, compatible with our recent report on the localization of its homolog in *Toxoplasma*^51^. However, *Ef*LPAAT2 exhibits an intriguing presence in the ER. These results suggest that the ER/Golgi complex cooperates with the apicoplast for the biogenesis of PtdOH and CDP-DAG.

Among downstream pathways, we identified *Ef*PIS and final enzymes of PtdCho and PtdEtn synthesis, *Ef*CEPT1 and *Ef*CEPT2. All these enzymes belong to the CDP-alcohol phosphotransferase family (Figure S3) and were located in the Golgi complex. CEPT is previously reported in the ER of *T. brucei*^52^, while PIS is localized in both ER and Golgi complex^53^. The expression of CEPT and PIS pathways in Golgi endows this organelle a significant role in lipid biogenesis of *E. falciformis*. PtdEtn synthesis by decarboxylating PtdSer through the catalytic action of PSD1 and PSD2 occurs in the mitochondrion and PV, respectively. Subcellular localizations of the two *Ef*PSD isoforms correspond to their homologs in *T*. *gondii*^54,55^, but different from *Pf*PSD residing in the ER^56^. Unlike kinetoplastids, where Kennedy pathways are the main source of PtdEtn^52,57^, PSD activity appears to play a major role in PtdEtn biogenesis in *T. gondii*^3,54,55^. The mitochondrion also harbors *Ef*PGPS and *Ef*CLS for the synthesis of PtdGro and cardiolipin that is similar to *T*. *brucei* where PGPS and CLS form a large complex in the inner mitochondrial membrane^58,59^. On the other hand, *Ef*PTS and *Ef*PSS are found in the ER/Golgi, which resembles the tachyzoites of *T*. *gondii*^7^. Most enzymes are likely to be essential for the sporozoite formation because unlike other infectious stages, this stage does not have access to host-derived metabolites. Physiological assessment of these proteins shall await the development of appropriate gene manipulation tools and in vitro culture for *E*. *falciformis*. Likewise, isotope labeling of sporulating oocysts will facilitate further research on de novo synthesis and functional evaluation of the eventual mutants.

Collectively, our study reveals a highly interweaved and compartmentalized network of lipid synthesis with varied phylogenetic origins and notable exceptions in *E*. *falciformis* sporozoites (Fig. 7). Such a complex network is expected to require a significant inter-organelle trafficking of lipids and their precursors. Multiple membrane contact sites have been reported between ER/Golgi complex, apicoplast and mitochondrion in apicomplexan parasites^60–62^, though it remains to be seen whether they serve as privileged zones of lipid exchange. Last but not least, this work rationalizes the evolutionary retention of an entire metabolic module in a model parasite that is meant to facilitate the development of free-living stage during the natural lifecycle.

## Materials and methods

### Biological reagents and resources

Oocysts of *E*. *falciformis* were obtained from Bayer AG (Leverkusen, Germany). The RHΔ*ku80*-Δ*hxgprt* strain of *T*. *gondii*^63^ was kindly provided by Vern Carruthers (University of Michigan, MI). The Δ*tgpts* mutant^7,20^ was generated by Ruben D. Arroyo-Olarte (Humboldt University Berlin, Germany). The human foreskin fibroblast (HFF) cells and NMRI mice were purchased from Cell Lines Service (Eppelheim, Germany) and Charles River Laboratories (Wilmington, MA), respectively. The yeast IPCS mutant (YPH499-HIS-GAL-AUR1) and corresponding expression vector (*pRS426-MET25*) were provided by Ralph T. Schwarz (University of Marburg, Germany). Primary antibodies recognizing the *Tg*F1B*, Tg*Fd, *Tg*GAP45, *Tg*GRA1, and *Tg*SAG1 proteins were provided by Peter Bradley (University of California, CA), Frank Seeber (Robert Koch Institute Berlin, Germany), Dominique Soldati-Favre (University of Geneva, Switzerland), Marie-France Cesbron-Delauw (University Joseph Fourier, France), and Jean-Francois Dubremetz (University of Montpellier, France), respectively. The antiserum recognizing *Eimeria tenella* (cross-reactive to *E. falciformis*) was donated by Fiona Tomley (Royal Veterinary College, UK). Antibodies against the HA, Myc and Ty1 epitopes were purchased from Sigma-Aldrich (St. Louis, MO). Secondary antibodies (Alexa488 and Alexa594) and oligonucleotides were procured from Life Technologies (Germany). The organelle markers in this study included *Tg*PTS-Myc for ER^7^, *Tg*Fd for apicoplast^64^, *Tg*ERD2-Ty1 for Golgi complex^65^, *Tg*F1B for mitochondrion^55^, *Tg*GAP45 for parasite periphery^66^, and *Tg*GRA1 for PV^67^.

### Propagation of *E. falciformis* and isolation of sporozoites

The lifecycle of *E*. *falciformis* was maintained by continuous passage of the parasite oocysts in NMRI mice as described elsewhere^22^. Oocysts in the animal feces were washed in water, floated with NaOCl, and stored in potassium dichromate at 4 °C up to 3 months^68^. The isolation of *E*. *falciformis* sporozoites was performed as described before^24^. In brief, purified oocysts were digested with 0.4% pepsin (pH 3, 37 °C, 1 h) before washing with PBS. Sporocysts were released by mixing oocysts with glass beads (0.5 mm) and vortexing in DMEM supplemented with 0.25% trypsin, 0.75% sodium tauroglycocholate, 20 mM glutamine, 100 units/ml penicillin, and 100 µg/ml streptomycin for 2 min at 37 °C. Free sporozoites were purified by DE-52 anion exchange chromatography^69^, and directly used for transfection or stored at −80 °C for lipidomic analysis, as stated below.

### Study approval

The propagation of *E. falciformis* in mouse was performed according to the German Animal Welfare Act (Tierschutzgesetz), as dictated by the overseeing authority Landesamt fuer Gesundheit und Soziales, Berlin, Germany (LAGeSo authorization # IC 113 – H 0098/04).

### In vitro culture of *T. gondii*

Tachyzoites of *T*. *gondii* were propagated by serial passage in HFF monolayers at a MOI of 3. HFFs were cultured in DMEM containing fetal calf serum (10%, PAN Biotech, Aidenbach, Germany), glutamine (2 mM), sodium pyruvate (1 mM), penicillin (100 units/ml), streptomycin (100 µg/ml) and MEM non-essential amino acids in a humidified incubator (5% CO_2_, 37 °C). Cells were harvested by trypsinization and grown to confluence in flasks, dishes or plates, as needed. For experiments, parasites were mechanically released from late-stage cultures and used immediately. Briefly, parasitized cells (40–42 h post infection) were scraped in fresh medium and squirted through 23 G and 27 G syringes (2× each) to obtain extracellular tachyzoites, which were either used directly for transfection and phenotyping assays, or stored at −80 °C for lipidomic analysis, as described below.

### Lipid analysis

Pellets of freshly frozen (metabolically quenched) purified sporozoites (*E*. *falciformis*) and tachyzoites (*T*. *gondii*) were suspended in 0.8 ml PBS (3 × 10^7^ parasites) and subjected to lipid extraction according to Bligh and Dyer^70^. Total lipids were dried under nitrogen stream and dissolved in 1 ml chloroform and methanol mixture (1:1), of which a 10 μl aliquot was introduced onto a Kinetex HILIC column (dimensions 50×4.6 mm, 2.6 μm, Phenomenex, Torrance, CA). Phospholipid (sub-)classes were resolved at a flow rate of 1 ml/min, as described earlier^71^. The column effluent was introduced into a LTQ-XL mass spectrometer (Thermo Scientific, Waltham, MA) and analyzed by electrospray ionization in positive and negative ionization modes. Commercial standards were used to calibrate and quantify the recovery of lipids. Acyl chain composition of individual lipids was determined by data-dependent MS/MS in separate runs. To confirm lipid class, acyl chain composition and elemental composition of the identified lipids, a subset of samples was run on a Fusion mass spectrometer (Thermo Scientific, Waltham, MA), operated at an orbitrap resolution of 120.000 and generating 30 data-dependent MS/MS spectra per second in the linear ion trap. For IPC structure studies, product ions were routed from the ion routing multipole to the orbitrap mass analyzer. Mass errors between observed lipid ions and theoretical *m/z* values were <2 ppm for orbitrap studies. Glycerophospholipids species were all from the diacyl subclass. We did not detect any ether-lipids. The procedure did not result in any noticeable lipid oxidation. The use of LCMS allowed us to discriminate two isobaric lipids of different classes (such as PtdEtn 36:2 and PtdCho 33:2), which were chromatographically fully separated. Data were processed using the most recent version of package “XCMS” under R (https://www.R-project.org)^72^. The lipid abbreviations herein follow the IUPAC-IUB nomenclature (http://www.chem.qmul.ac.uk/iupac/lipid/).

### Bioinformatics and phylogeny studies

Enzymes of phospholipid biogenesis in protozoan parasites were identified using EuPathDB (www.eupathdb.org) and ToxoDB (www.toxodb.org)^26,73^. All sequences reported in this study were experimentally annotated. The functional domains and transmembrane regions were predicted using the Simple Modular Architecture Research Tool (SMART) (http://smart.embl-heidelberg.de) and Transmembrane Hidden Markov Model (TMHMM) (http://www.cbs.dtu.dk/services/TMHMM/), respectively. The secretory signal or mitochondrial targeting peptide was examined using SignalP v4.1 (http://www.cbs.dtu.dk/services/SignalP/) and MitoProt (https://ihg.gsf.de/ihg/mitoprot.html) algorithms. Phylogenetic trees were constructed by CLC Sequence Viewer v7.7 (http://www.clcbio.com/products/clc-sequence-viewer/) and visualized with FigTree v1.4.2 (http://tree.bio.ed.ac.uk/software/figtree/) softwares. The sequence accession numbers with respective organism names are described in Table S2.

### Molecular cloning and making of transgenic parasites

RNA was isolated from freshly purified sporozoites of *E. falciformis* using TRIzol-extraction method and subsequently reverse-transcribed into first-strand cDNA (Life Technologies, Germany). The genomic DNA was isolated using the genomic DNA preparation kit (Jena Bioscience, Jena, Germany). All amplicons were amplified using Pfu Ultra II fusion HS polymerase (Agilent Technologies, Santa Clara, CA) and specific primers (Table S3). For ectopic overexpression of selected proteins in *E. falciformis*, the 5′ and 3′ UTRs of *Ef*HIS4 and the ORF of *Ef*PIS were cloned into the *pTKO-DHFR-TS* plasmid by overlap extension PCR. The ORFs of other enzymes were cloned into the same vector by replacing *Ef*PIS ORF using *Avr*II/*Pac*I restriction sites. For ectopic overexpression of selected proteins in *T. gondii* tachyzoites, ORFs of *E. falciformis* sporozoites were cloned into the *pGRA1-UPKO* plasmid using *Nsi*I/*Pac*I restriction sites. The eventual constructs were transformed into *E. coli* XL-1b strain for cloning and vector amplification.

The constructs were transfected into freshly released *T. gondii* tachyzoites of the RHΔ*ku80*-Δ*hxgprt* strain (50 μg DNA, ~10^7^ parasites) or *E. falciformis* sporozoites (50 μg DNA, 2 × 10^6^ parasites) suspended in filter-sterile cytomix (120 mM KCl, 0.15 mM CaCl_2_, 10 mM K_2_HPO_4_/KH_2_PO_4_, 25 mM HEPES, 2 mM EGTA, 5 mM MgCl_2_, 5 mM glutathione, 5 mM ATP, pH 7.6) using a BTX electroporation instrument (2 kV, 50 Ω, 25 μF, 250 μs). Tachyzoites expressing ER-localized (*Ef*G3PAT1, *Ef*LPAAT2, *Ef*CDS1, *Ef*PTS) and Golgi-localized (*Ef*LPAAT1, *Ef*PSS, *Ef*CEPT1, *Ef*CEPT2, *Ef*PIS) proteins were co-transfected with constructs encoding for *Tg*PTS-Myc and *Tg*ERD2-Ty1, respectively, for co-localization studies. Transfected parasites were used to infect HFFs for indirect immunofluorescence assays (see below), and screened for transient expression of *E. falciformis* enzymes. The Δ*tgpts*/*Ef*PTS strain was generated by transforming the *pGRA1-UPKO-EfPTS* construct into the Δ*tgpts* strain followed by negative selection for the disruption of the *TgUPRT* locus using 5-fluorodeoxyuridine (5 μM)^74^.The drug-resistant parasites were cloned by limiting dilution and screened by PCR for the expression of *Ef*PTS. Positive clones were subjected to the lytic cycle assays and lipidomic analysis, as described elsewhere.

### Indirect immunofluorescence assays

Parasitized HFFs cultured on glass coverslips were washed with PBS 20–24 h (tachyzoites) or 40–42 h (sporozoites) post infection, fixed with 4% paraformaldehyde for 10 min, and neutralized with 0.1 M glycine in PBS for 5 min. Cells were permeabilized with 0.2% triton X-100 in PBS for 20 min and treated with 2% bovine serum albumin in 0.2% triton X-100 and PBS for 20 min. Samples were stained with a combination of primary antibodies (anti-HA, 1:3000; anti-Myc, 1:3000; anti-Ty1, 1:50; anti-*Tg*Fd, 1:500; anti-*Tg*F1B, 1:1000; anti-*Tg*GRA1, 1:500; anti-*Tg*GAP45, 1:3000; anti-*Tg*SAG1, 1:1500; antiserum recognizing *E. tenella*, 1:2000) for 1 h, as shown in figures. Cells were washed three times with 0.2% triton X-100 in PBS and then stained with Alexa488/594-conjugated antibodies for 45 min. Following three additional washings with PBS, samples were mounted in fluoromount G / DAPI mixture (Southern Biotech, Birmingham, AL), and then stored at 4 °C. Imaging was done by a fluorescence microscope (ApoTome, Zeiss, Germany).

### Lytic cycle assays

Plaque assays were performed by infecting HFF monolayers grown to confluence in 6-well plates (250 tachyzoites/well). Infected cultures were incubated unperturbed for 7 days and samples were fixed with ice-cold methanol followed by staining with crystal violet dye. Plaques were imaged and scored for sizes and numbers using the ImageJ software (NIH, Bethesda, MD). To test the invasion efficiency, tachyzoites of each strain were used to infect confluent HFFs (MOI, 10) for 1 h. Noninvasive parasites were stained with anti-*Tg*SAG1 antibody prior to detergent permeabilization. Cells were then washed 3 times with PBS, permeabilized with 0.2% triton X-100 in PBS for 20 min and then stained with anti-*Tg*GAP45 antibody to visualize intracellular parasites. The percentages of invaded parasites were determined to compare the invasion efficiency of different strains. For replication and egress assays, confluent HFFs cultured on coverslips in 24-well plates were infected with tachyzoites of each strain (MOI, 1), fixed at indicated time points (40 h for replication; 48 and 72 h for egress) and then subjected to immunostaining using anti-*Tg*GAP45 antibody, as described above. The mean percentage of vacuoles containing variable numbers of intracellular parasites was scored to examine the replication phenotype. Egress was calculated by comparing the vacuole numbers between 48 h (intracellular) and 72 h (egressing). For motility assays, fresh syringe-released parasites were incubated on BSA (0.01%)-coated coverslips in Hank’s Balanced Salt Solution (15 min, 37 °C), fixed with 4% paraformaldehyde and 0.05% glutaraldehyde (10 min), and then stained with anti-*Tg*SAG1 and Alexa488 antibodies. Motile fractions and trail lengths were quantified using ImageJ software.

### Functional complementation of yeast

Yeast cells (YPH499-HIS-GAL-AUR1) were grown in synthetic histidine-free minimal medium (0.67% yeast nitrogen base; Difco) supplemented with appropriate amino acids, 4% galactose and 2% raffinose (SGR medium). The mutant was transformed with the constructs expressing *Ef*SLS, *Tg*SLS, *Sc*IPCS, or the empty vector (*pRS426-MET25*). The cDNAs of *Ef*SLS and *Tg*SLS were amplified from the total RNA isolated from *Eimeria* sporozoites and *Toxoplasma* tachyzoites, respectively. *Sc*IPCS ORF was cloned from yeast gDNA (primers in Table S3). The ORFs were ligated at the *Spe*I and *Hind*III into *pRS426-MET25* vector expressing the *URA3* gene for selecting yeast transformants in uracil-deficient medium. The plasmid allowed the expression of a protein of interest under the control of the *MET25* promoter of *S. cerevisiae*. Transformation was performed using standard yeast protocols. Transgenic cells were grown in uracil- and histidine-dropout SGR medium at 30 °C, and positive clones were identified by PCR and sequencing. For complementation assays, yeast cells were grown in liquid medium to an *A*_600_ of ∼0.1, and serial dilutions (1:5) were spotted on synthetic uracil- and histidine-dropout plates containing either 4% galactose and 2% raffinose (synthetic galactose-raffinose or SGR medium), or 2% glucose (synthetic dextrose or SD medium). Plates were incubated for 3–4 days at 30 °C.

### Statistics

All data are shown as the mean with SEM from three or six independent assays, as indicated in figure legends. Statistical analyses were performed using the GraphPad Prism program. Significance was tested by unpaired two-tailed Student’s *t* test with equal variances (*p < 0.05, **p < 0.01, ***p < 0.001).

### Data deposition

Sequences of *Eimeria falciformis* proteins reported in this study have been deposited to GenBank. *Ef*G3PAT1, KX785365; *Ef*G3PAT2, KX785366; *Ef*LPAAT1, KX785367; *Ef*LPAAT2, KX785368; *Ef*CDS1, KX017547; *Ef*CDS2, KX017548; *Ef*PAP, KX785369; *Ef*DGK1, KX785370; *Ef*DGK2, KX785371; *Ef*DGK3, KX785372; *Ef*PGPS, KX7853713; *Ef*CLS, KX785374; *Ef*PIS, KX785375; *Ef*CEPT1, KX785376; *Ef*CEPT2, KX785377; *Ef*CEPT3, KX785378; *Ef*PSD1, KX785379; EfPSD2, KX785380; *Ef*PSS, KX785381; *Ef*PTS, KX785382; *Ef*SLS, MF577034.

## Electronic supplementary material

Supporting Figure S1-S5(PDF 623 kb)

Supporting Tables 1-3(XLSX 520 kb)

Supplementary Information
