# Supplementary figures and images for: Lipid analysis of Eimeria sporozoites reveals exclusive phospholipids, a phylogenetic mosaic of endogenous synthesis, and a host-independent lifestyle
Source: Cell Discov. 2018 May 22;4:24. doi: 10.1038/s41421-018-0023-4 (PMC5964319; doi:10.1038/s41421-018-0023-4)

Figure S1

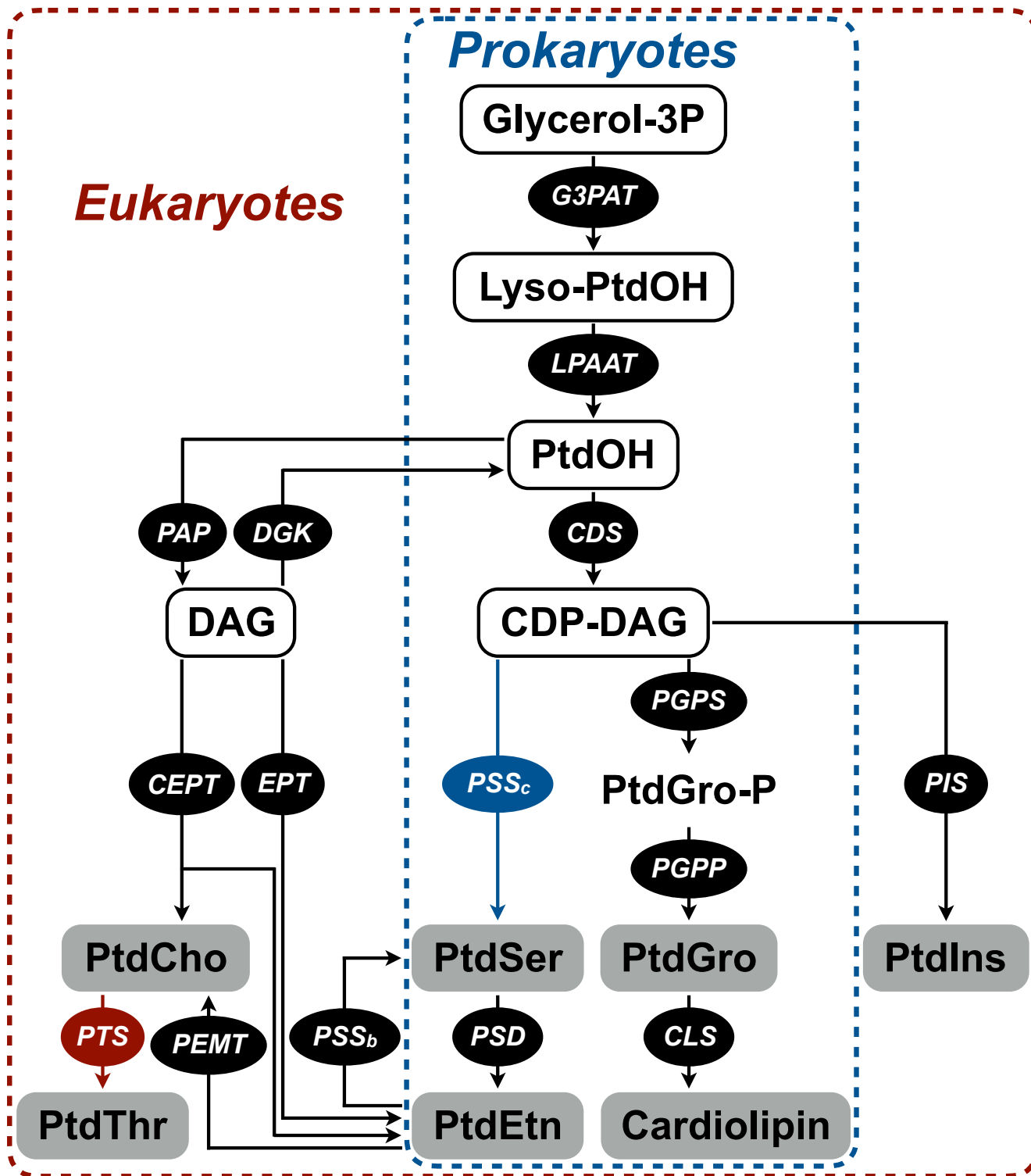

**Figure S2**

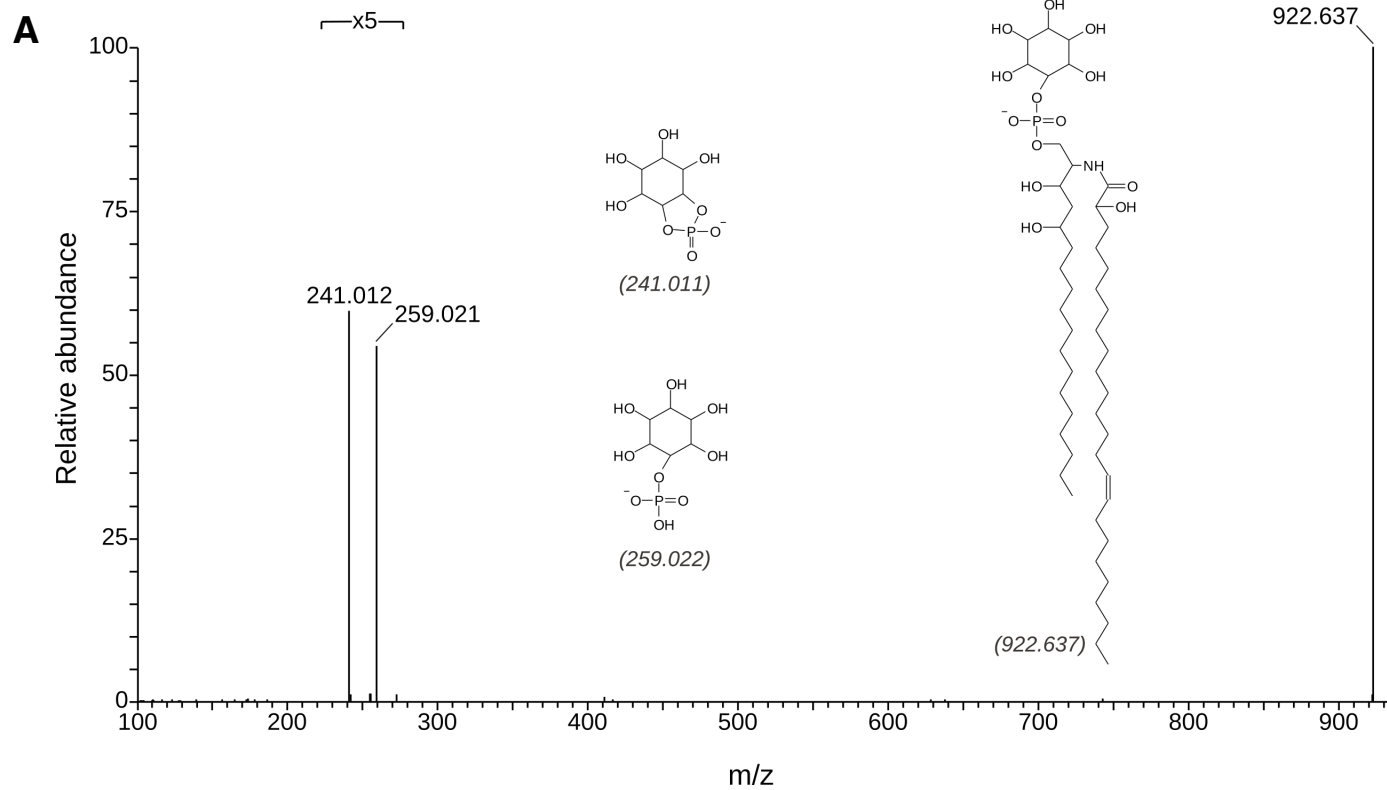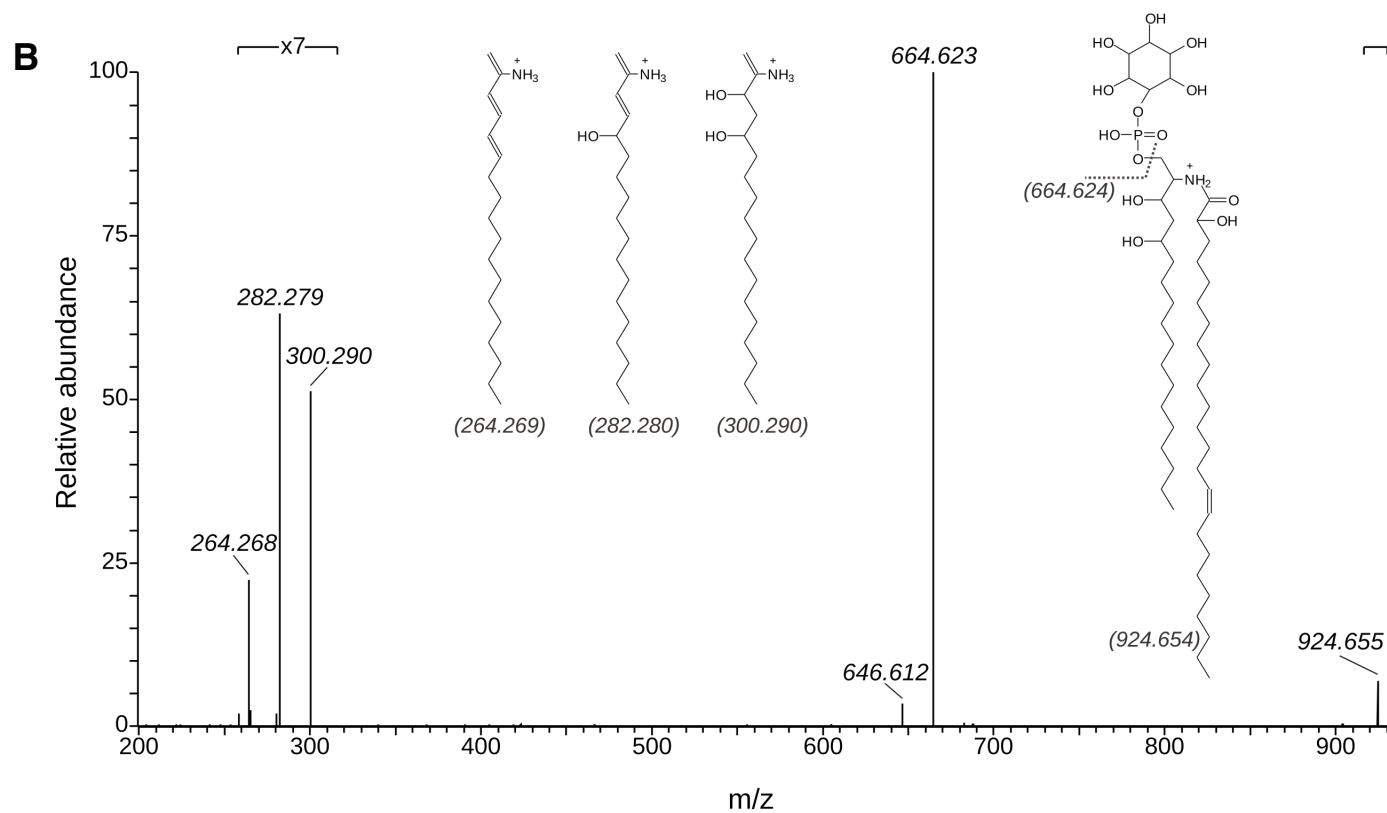

**Figure S3**

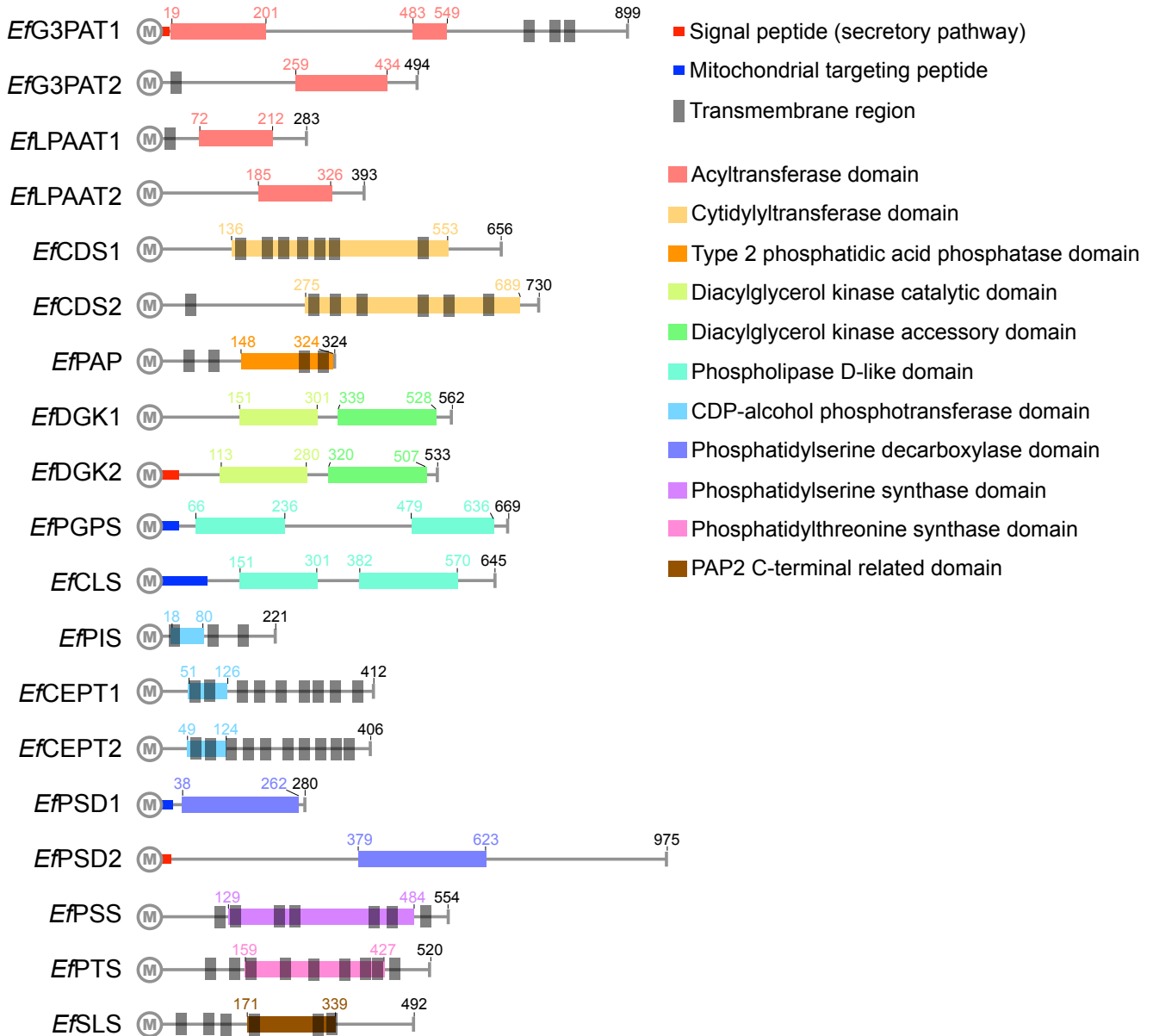

Figure S4

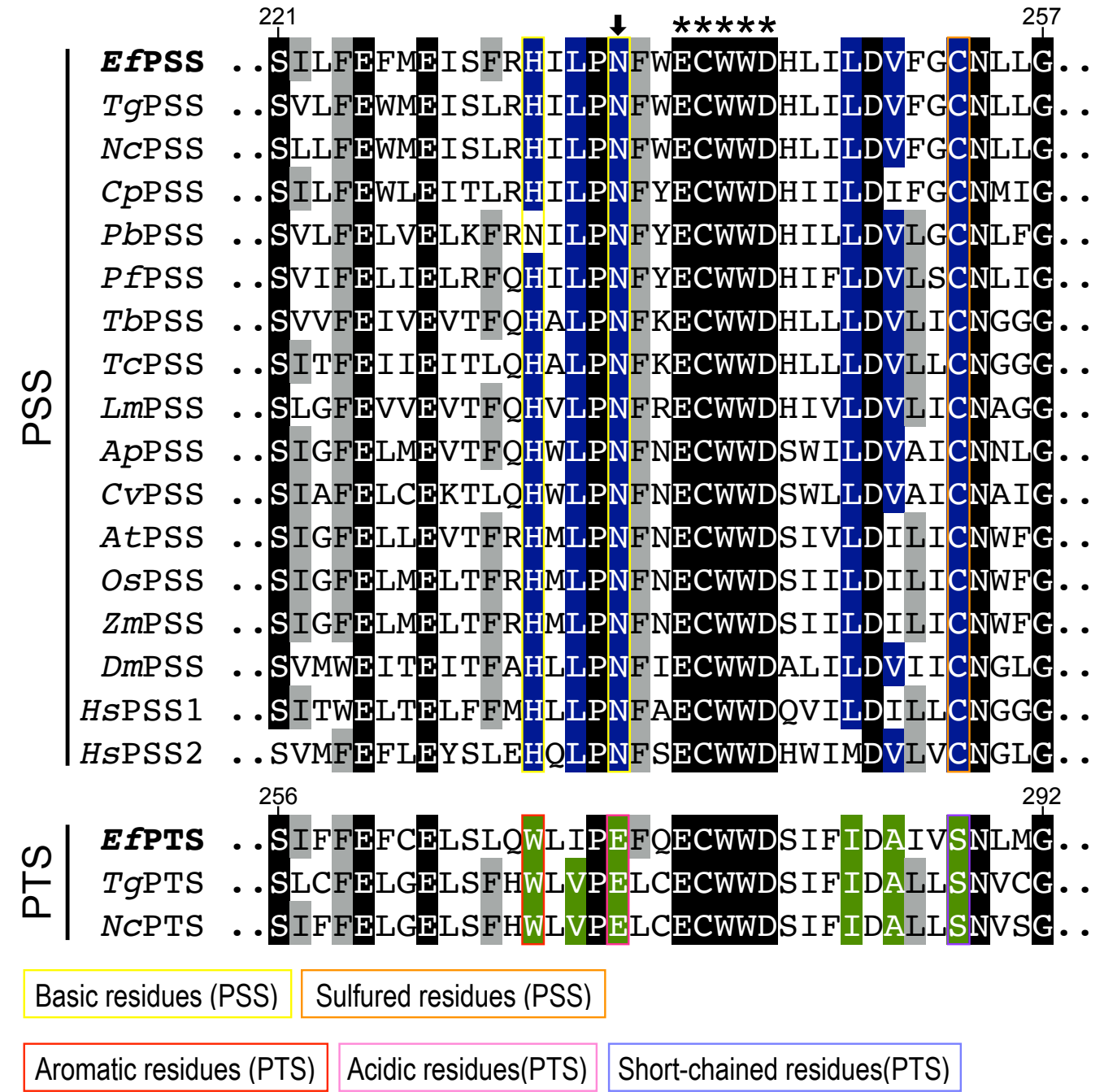

**Figure S5**

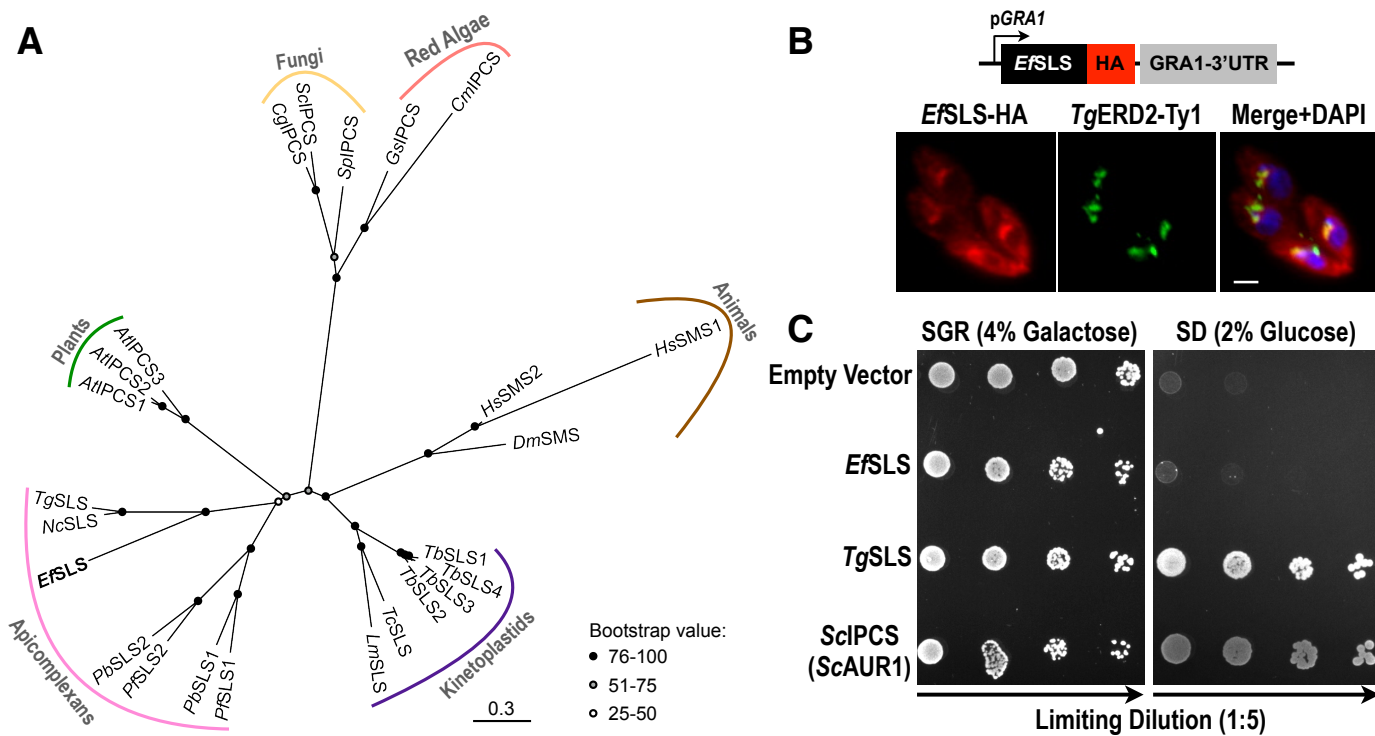

Supplement: Supplementary file 1 — Supporting Figure S1-S5(PDF 623 kb) [file 41421_2018_23_MOESM1_ESM.pdf]
