# Supplementary material for: Lipid analysis of Eimeria sporozoites reveals exclusive phospholipids, a phylogenetic mosaic of endogenous synthesis, and a host-independent lifestyle
Source: Cell Discov. 2018 May 22;4:24. doi: 10.1038/s41421-018-0023-4 (PMC5964319; doi:10.1038/s41421-018-0023-4)
Supplement: Supplementary file 3 — Supplementary Information [file 41421_2018_23_MOESM3_ESM.docx]

**Supplementary Information**

**Figure S1** Synthesis of key glycerophospholipids in eukaryotes and prokaryotes. The pathways with black arrows in the red box are present in mammalian cells (human/mouse), whereas bacteria harbor only the part of the network in the blue box, including PSS_c_ (blue). PTS (red) was recently identified as an exclusive enzyme in the coccidian parasite *T. gondii*. Major phospholipids, intermediates of synthesis and underlying enzymes are shown in gray, white and black backgrounds, respectively. *Abbreviations*: CDP-DAG, cytidine diphosphate-diacylglycerol; CDS, cytidine diphosphate-diacylglycerol synthase; CEPT, choline/ethanolamine phosphotransferase; CLS, cardiolipin synthase; DAG, diacylglycerol; DGK, diacylglycerol kinase; EPT, ethanolamine phosphotransferase; G3PAT, glycerol 3-phosphate acyltransferase; Glycerol-3P, glycerol 3-phosphate; LPAAT, lysophosphatidic acid acyltransferase; Lyso-PtdOH, lysophosphatidic acid; PAP, phosphatidic acid phosphatase; PEMT, phosphatidylethanolamine N-methyltransferase; PGPP, phosphatidylglycerol phosphate phosphatase; PGPS, phosphatidylglycerol phosphate synthase; PIS, phosphatidylinositol synthase; PSD, phosphatidylserine decarboxylase; PSS_b_, phosphatidylserine synthase (base-exchange type); PSS_c_, phosphatidylserine synthase (CDP-DAG-dependent); PtdCho, phosphatidylcholine; PtdEtn, phosphatidylethanolamine; PtdGro, phosphatidylglycerol; PtdGro-P, phosphatidylglycerol phosphate; PtdIns, phosphatidylinositol; PtdOH, phosphatidic acid; PtdSer, phosphatidylserine; PtdThr, phosphatidylthreonine; PTS, phosphatidylthreonine synthase.

**Figure S2** Mass spectrometric fragmentation of IPC. MS/MS analysis of a tentative IPC ion confirms the presence of a hexose-phosphate head-group in the negative ionization mode **(A)**, as well as a phytosphingosine and α-hydroxylated fatty acyl group in the positive ionization mode **(B)**. The structure of the precursor ion and the most prominent product ion are given together with their theoretical masses.

**Figure S3** Primary structures of putative enzymes involved in phospholipid synthesis in the sporozoites of *E. falciformis*. Predicted functional domains, transmembrane regions, as well as signal and mitochondrial targeting peptides are shown with boxes in different colors, as indicated on the right. The numbers represent the positions of the functional domains and the lengths of the experimentally annotated open reading frames. The length of domains and proteins are all shown to a relative scale. For phospholipid synthesis pathways and related enzymes in other parasites, see Figure S1 and Table S1. Our searches also found the choline/ethanolamine kinases and other intermediate enzymes of the Kennedy pathway. However, only the last enzymes of each pathway catalyzing the lipid synthesis are shown here.

**Figure S4** Multiple alignments of the conserved motifs present in base-exchange-type PSS and PTS from selected organisms. The residues identical across all sequences are shaded with black color, while those only conserved in PSS or PTS are highlighted in blue or green, respectively. The putative catalytic site (ECWWD) is marked with stars on top of the alignments, which was deleted in the Δ*tgpts* mutant (see Figure 5A). The substrate-binding residue is highlighted with a black arrow. Sequence information including accession numbers and organism names are listed in Table S2.

**Figure S5** Characterization of sphingolipid synthase from *E. falciformis*. **(A)** Phylogenetic tree depicting the evolutionary relationships of *Ef*SLS with orthologs from indicated organisms. Circles on the branches indicate the bootstrap values for parsimony. Sequence information including accession numbers and the organism names are described in Table S2. **(B)** Localization of C-terminally HA-tagged *Ef*SLS in the tachyzoites of *T. gondii*. A construct overexpressing *Ef*SLS-HA under the control of the *GRA1* elements was transfected in tachyzoites followed by fixation (24 h post-infection), and staining with anti-HA and anti-Ty1 antibodies. Immunostaining of *Tg*ERD2-Ty1 shows the Golgi network. **(C)** Complementation by heterologous expression of *Ef*SLS and *Tg*SLS in the AUR1 mutant of *S. cerevisiae* (YPH499-HIS-GAL-AUR1). *Ef*SLS and *Tg*SLS were expressed under the control of the *MET25* promoter. Empty *pRS426-MET25*, and *Sc*IPCS (*Sc*AUR1) expression vectors were also included as negative and positive controls, respectively. While all transgenic strains grew in permissive SGR medium (galactose as a carbon source) as expected, only *Tg*SLS and *Sc*IPCS were able to restore the growth of the yeast mutant in non-permissive SD medium (glucose as a carbon source).

**Table S1** EuPathDB accession numbers of the enzymes involved in lipid biogenesis in selected protozoan parasites.

**Table S2** Sequences used for phylogenetic analysis. The parasite sequences are shown with EuPathDB accession numbers, while others are listed with NCBI accession numbers.

**Table S3** Oligonucleotides used in this study.
